# Supplementary material for: Non-linear associations of a body shape index with diabetes among adults: A cross-sectional study
Source: PLoS One. 2025 Oct 23;20(10):e0333883. doi: 10.1371/journal.pone.0333883 (PMC12548853; doi:10.1371/journal.pone.0333883)
Supplement: Attachment — Submitted filename: Response to reviewers.docx [file pone.0333883.s001.docx]

Re: Response for manuscript PONE-D-25-02776 “Non-linear associations of a body shape index with diabetes among adults: a cross-sectional study”

Dear Editor, Dear reviewers

We appreciate your letter dated 3 June , 2025.

We are gratified to have received the peer review comments from Frontiers in Psychiatry. Our sincere thanks go to the editors and reviewers for their invaluable support and guidance in refining our work. We deeply appreciate your constructive feedback and insightful suggestions regarding our paper.

Adhering to the guidelines provided in your letter, we have uploaded both the revised manuscript and a version with tracked changes. All modifications are prominently marked in red font for easy identification.

Enclosed with this letter is our detailed, point-by-point response to the reviewers' comments. We are grateful for the opportunity to resubmit our revised manuscript. It is our sincere hope that Frontiers in Psychology will consider our manuscript suitable for publication.

Sincerely,

The authors

Encl. Responses to the comments from Reviewers 1 and Reviewers 2.

Reply to Reviewers 1

Dear Reviewers,

Thank you for the time and effort you have dedicated to reviewing our manuscript and for your constructive feedback and valuable suggestions.

We greatly appreciate your detailed comments, which have been instrumental in enhancing the quality of our manuscript. We believe that our responses have adequately addressed all of your concerns. In this letter, we will individually address each of your comments and provide our corresponding responses.

We sincerely hope that the revisions made to our manuscript align with your expectations and look forward to your favorable consideration.

Sincerely,

The authors

**Major comments:**
1. The manuscript often implies causality ("ABSI increases diabetes risk"), which is not appropriate for a cross-sectional study. Please revise these statements throughout to reflect association, not causation.

**Author's Response:**

We appreciate the reviewer’s insightful comment. In response, we have carefully revised the manuscript to avoid any causal language and to reflect the associative nature of our cross-sectional study. Specifically, phrases such as “increased risk” and “leads to” have been replaced with non-causal alternatives such as “associated with,” “higher odds of,” or “linked to.” These changes have been made throughout the Abstract, Results, and Discussion sections to ensure alignment with the study design.

**Modifications:**

1)Original sentence: The two-part linear regression model demonstrated that when ABSI is less than or equal to 9.54, each unit increase in ABSI is associated with an 87% increase in the risk of diabetes.

Revised sentence (Line 212-215): The two-part linear regression model demonstrated that when ABSI is less than or equal to 9.54, each unit increase in ABSI is associated with an 87% increased likelihood of diabetes.

2)Original sentence: This study employed multivariate linear regression analysis to evaluate the association between ABSI and the risk of diabetes.

Revised sentence (Line 20-22): This study employed multivariate linear regression analysis to evaluate the association between ABSI and the likelihood of having diabetes.

3)Original sentence: Participants in the highest quartile of ABSI had a 96% greater risk of diabetes.

Revised sentence (Line 28-29): Participants in the highest quartile of ABSI had a 96% higher odds of having diabetes.

4)Original sentence: Each unit increase in ABSI score is associated with a 42% increase in the risk of diabetes.

Revised sentence (Line 195-196): Each unit increase in ABSI score is associated with a 42% increase in the likelihood of diabetes.

5)Original sentence: When ABSI was categorized into quartiles, participants in the highest quartile of ABSI had a 96% increased risk of diabetes compared to those in the lowest quartile.

Revised sentence (Line 197-199): When ABSI was categorized into quartiles, participants in the highest quartile of ABSI had 96% higher odds of having diabetes compared to those in the lowest quartile.

6)Original sentence: Our results indicate that each unit increase in ABSI is associated with a 42% to 66% increase in the risk of diabetes.

Revised sentence (Line 283-285): Our results indicate that each unit increase in ABSI is associated with 42% to 66% higher odds of having diabetes.

7)Original sentence: The highest quartile group exhibited nearly a 96% increased risk of diabetes compared to the lowest quartile group.

Revised sentence (Line 286-288): The highest quartile group were nearly 96% more likely to have diabetes compared to the lowest quartile group.

8)Original sentence: These results underscore the potential of ABSI as an independent predictor of diabetes risk.

Revised sentence (Line 288-289): These results underscore the potential of ABSI as an independent marker associated with diabetes.

2. The conclusion should explicitly state that longitudinal studies are needed to explore causality.

**Author's Response:**

Thank you for the valuable suggestion. We have revised the conclusion to explicitly state that longitudinal studies are needed to explore the temporal and causal relationship between ABSI and diabetes. The updated conclusion also highlights the limitations of the cross-sectional design and the need for further research in diverse populations.

**Modifications:**

Line 450-454：

However, given the cross-sectional nature of the study and the possibility of residual confounding, causality cannot be inferred. Future longitudinal studies are needed to confirm the temporal sequence and explore causal relationships between ABSI and diabetes, as well as to assess the applicability of these findings across diverse ethnic and cultural groups.

3. Threshold Definition & Interpretation
**Author's Response:**

Thank you for your valuable comment. In response, we have revised the manuscript to provide a clearer definition and interpretation of the threshold identified in our two-piece linear regression model. We now explicitly define the threshold as the ABSI value at which the association with diabetes risk changes significantly and describe the two-step recursive method used to determine it. Additionally, we clarify the rationale for using a segmented model and the interpretation of the inflection point in both statistical and clinical terms. These revisions have been made to improve clarity and transparency in the methodology and results interpretation.

**Modifications:**

Line 146-159：

To examine whether a threshold effect exists in the association between ABSI and diabetes, we used a two-piece linear regression model with a smoothing function. A threshold, or inflection point, was defined as the value of ABSI at which the relationship with diabetes risk changes significantly—statistically identified as the point where segmented regression yields the best model fit [20, 21]. The threshold was determined using a two-step recursive approach [20, 21]. In the first step, we screened candidate thresholds across the 5th to 95th percentiles of ABSI (at 5% intervals) and selected the percentile that maximized the log-likelihood of the model. In the second step, we narrowed the range around the initial estimate and applied an iterative procedure to identify the precise ABSI value that yielded the highest model likelihood. To compare the goodness of fit between the segmented (two-piece) and standard linear models, we performed a log-likelihood ratio test. A statistically significant result from this test was used as the criterion to support the presence of a threshold effect.

4. The choice of the ABSI threshold (e.g., 9.54) is automatically selected, but the method is not explained in enough detail. Please clarify how the inflection point was determined, and why the model fit was better with a two-piece model (e.g., include AIC/BIC values or R² comparison).
**Author's Response:**

Thank you for your constructive comment. We have revised the Methods section to provide a more detailed explanation of how the ABSI threshold was determined using a two-step recursive procedure based on log-likelihood optimization. Although AIC, BIC, or R² values were not available, we used a log-likelihood ratio test to compare model fit between the segmented and standard linear models. This methodological clarification has been incorporated without introducing any result-based interpretation in the Methods section.

**Modifications:**

Line 146-159

To examine whether a threshold effect exists in the association between ABSI and diabetes, we used a two-piece linear regression model with a smoothing function. A threshold, or inflection point, was defined as the value of ABSI at which the relationship with diabetes risk changes significantly—statistically identified as the point where segmented regression yields the best model fit. The threshold was determined using a two-step recursive approach. In the first step, we screened candidate thresholds across the 5th to 95th percentiles of ABSI (at 5% intervals) and selected the percentile that maximized the log-likelihood of the model. In the second step, we narrowed the range around the initial estimate and applied an iterative procedure to identify the precise ABSI value that yielded the highest model likelihood. To compare the goodness of fit between the segmented (two-piece) and standard linear models, we performed a log-likelihood ratio test. A statistically significant result from this test was used as the criterion to support the presence of a threshold effect.

5. Explain what the ABSI value of 9.54 represents in practical or clinical terms—e.g., how does this relate to real-world body measurements?

**Author's Response:**

Thank you for your valuable comment. In response, we have added an explanation of the clinical relevance of the ABSI threshold of 9.54 in the Discussion section. Specifically, we clarified that this value approximately corresponds to a waist circumference of 106.35 cm in an average adult with a height of 1.70 m and a BMI of 25 kg/m², thereby improving the practical interpretability of our findings.

**Modifications:**

Line 336-346

This study also identified a non-linear relationship between ABSI and diabetes, manifesting as a distinct inverse L-shaped curve. Through smooth curve fitting and threshold effect analysis, the inflection point of ABSI was determined to be 9.54. Below this inflection point, each unit increase in ABSI was associated with an 87% higher odds of diabetes, whereas above this point, the association was no longer statistically significant. It is important to note that an ABSI value of 9.54, based on the formula incorporating waist circumference, height, and BMI, approximately corresponds to a waist circumference of 106.35 cm in an average adult with a height of 1.70 m and a BMI of 25 kg/m². This threshold may serve as a practical reference point for identifying individuals at elevated diabetes risk due to central adiposity, even when BMI alone does not appear abnormal.

6. While subgroup analyses (stroke, gender, race) are reported, interaction terms are mentioned only briefly. Please elaborate on:How interaction p-values were calculated. Whether these were pre-specified or exploratory.

**Author's Response:**

Thank you for your thoughtful comment. We have revised the Methods section to provide additional details regarding the interaction analysis. Specifically, we now clarify that interaction terms between ABSI and each stratification factor (e.g., gender, race, stroke, hypertension) were included in multivariable logistic regression models. P-values for interaction were derived using Wald tests. We also note that these analyses were exploratory and not pre-specified in the original study design. These clarifications have been incorporated to improve the transparency of our analytic approach.

**Modifications:**

Line 160-169

Finally, the stability of the primary outcomes was explored through multifactorial stratified subgroup analysis, with stratification factors including gender (male/female), race (Non-Hispanic White/Non-Hispanic Black/Mexican American/Other Hispanic/Other), hypertension (yes/no), and stroke (yes/no). To formally assess effect modification, interaction terms between ABSI and each stratification factor (e.g., ABSI × gender) were included in the multivariable logistic regression models. P-values for interaction were calculated using Wald tests for the corresponding interaction terms. These analyses were exploratory in nature and were not pre-specified in the original study design.

7. The biological plausibility behind the significant interaction with stroke.

**Author's Response:**

Thank you for this valuable comment. In response, we have revised the Discussion section to elaborate on the potential biological mechanisms underlying the observed interaction between ABSI and stroke. Specifically, we now discuss how stroke-related metabolic changes—such as increased insulin resistance, chronic inflammation, and altered body composition—may enhance the impact of central adiposity on diabetes risk. These additions aim to strengthen the biological plausibility of our findings and highlight the importance of considering comorbid conditions in ABSI-based risk assessment.

**Modifications:**

Line 389-339

Notably, our results identified a significant interaction between ABSI and stroke status, indicating that the association between ABSI and diabetes risk differs between individuals with and without a history of stroke. Specifically, among participants with a history of stroke, the relationship was more pronounced. This may be attributed to stroke-related alterations in metabolic function, such as increased insulin resistance, chronic low-grade inflammation, and changes in body composition due to reduced mobility or muscle mass loss. These factors may amplify the impact of central adiposity, as reflected by ABSI, on diabetes risk. The presence of this interaction underscores the importance of considering comorbid conditions such as stroke when evaluating the clinical utility of ABSI in diabetes risk assessment and disease management.

8. Figures 2 and 3 need clearer legends, especially explaining what the lines and ribbons represent. Adding sample sizes, axis labels, and whether curves are adjusted or crude is important.

**Author's Response:**

Thank you for this helpful comment. We have revised the legends of Figures 2 and 3 to clearly describe the meaning of the lines and shaded areas, indicating whether the curves are based on adjusted or unadjusted models. Axis labels have also been clarified to specify the variables plotted and their interpretation, and sample sizes have been added for relevant subgroups. Additionally, we have converted all figures to high-resolution TIFF format to improve image clarity and ensure publication-quality presentation.

**Modifications:**

Line 221-225

Fig. 2 Adjusted association between ABSI and the predicted probability of diabetes. The red and blue curves represent multivariable logistic regression estimates with 95% confidence intervals, derived from unweighted data. Models were adjusted for age, gender, race, education level, smoking status, alcohol intake, hypertension, and stroke. X-axis: ABSI (A Body Shape Index); Y-axis: predicted probability of diabetes. Sample size: n = 34,693.

Line 243-249

Fig. 3 Adjusted association between ABSI and the probability of diabetes, stratified by gender. The red and blue smooth curves represent the estimated diabetes probabilities for males and females, respectively, based on multivariable models adjusted for sex, age, family poverty income ratio, race, education, hypertension, Marital status, Alcohol, Arthritis, Congestive Heart Failure, Coronary Heart Disease, Angina, Heart Attack, Stroke, and HDL cholesterol.. The Y-axis indicates the predicted probability of diabetes; the X-axis represents ABSI (A Body Shape Index). Sample sizes: male group (n = 17,577), female group (n = 17,116).

10. Table 2 and Table 4 are difficult to read due to formatting. Consider splitting or simplifying tables and ensuring that all model covariates are defined in footnotes.

**Author's Response:**

Thank you for your valuable suggestion. We have reformatted Tables 2 and 4 to improve readability by simplifying their structure and layout. Additionally, we have included detailed footnotes to define all covariates used in the regression models for clarity and completeness.

**Modifications:**

Line 201

**Table 2.** Association between A Body Shape Index and diabetes.

| **Variables** | **Model 1^1^** | | | **Model 2^2^** | | | **Model 3^3^** | | |
| --- | --- | --- | --- | --- | --- | --- | --- | --- | --- |
| **ABSI** | **β** | **95% CI** | **P value** | **β** | **95% CI** | **P value** | **β** | **95% CI** | **P value** |
| **Continuous** | 1.66 | 1.59, 1.74 | **<0.001** | 1.64 | 1.54, 1.73 | **<0.001** | 1.42 | 1.33, 1.52 | **<0.001** |
| **Categories** | **OR** | **95% CI** | **P value** | **OR** | **95% CI** | **P value** | **OR** | **95% CI** | **P value** |
| Tertile 1 | 1.0 |  |  | 1.0 |  |  | 1.0 |  |  |
| Tertile 2 | 1.75 | 1.57, 1.96 | **<0.001** | 1.45 | 1.28, 1.64 | **<0.001** | 1.22 | 1.05, 1.41 | **0.007** |
| Tertile 3 | 2.30 | 2.07, 2.56 | **<0.001** | 1.842 | 1.63, 2.08 | **<0.001** | 1.42 | 1.23, 1.63 | **<0.001** |
| Tertile 4 | 3.18 | 2.87, 3.53 | **<0.001** | 2.71 | 2.40, 3.06 | **<0.001** | 1.96 | 1.69, 2.26 | **<0.001** |
| P for trend | **0.004** | | | **0.019** | | | **0.032** | | |

**^1^**Non-adjusted model I adjust for: None.

**^2^**Adjust II model adjust for: sex, age, family poverty income ratio, race, education.

**^3^**Adjust III model adjust for: sex, age, family poverty income ratio, race, education, hypertension, Marital status, Alcohol, Arthritis, Congestive Heart Failure, Coronary Heart Disease, Angina, Heart Attack, Stroke, and HDL cholesterol.

Line 251

**Table 4.** Threshold effect analysis of ABSI on diabetes using a two-piecewise linear regression model.

|  | **Male** | | | **Female** | | |
| --- | --- | --- | --- | --- | --- | --- |
| **Model I**^1^ | **Adjusted OR^3^** | **95%CI^4^** | **P value** | **Adjusted OR** | **95%CI** | **P value** |
| One line slope | 1.53 | 1.41, 1.67 | **<0.001** | 1.470 | 1.36, 1.59 | **<0.001** |
| **Model II**^2^ | **Adjusted OR** | **95%CI** | **P value** | **Adjusted OR** | **95%CI** | **P value** |
| Turning point (K) | 9.43 | | | 9.29 | | |
| < K | 1.84 | 1.63, 2.08 | **<0.001** | 2.07 | 1.77, 2.42 | **<0.001** |
| > K | 0.99 | 0.78, 1.25 | 0.90 | 1.08 | 0.93, 1.25 | 0.32 |
| OR  between < 9.54 and > 9.54 | 0.54 | 0.39, 0.73 | **<0.001** | 0.52 | 0.40, 0.67 | **<0.001** |
| Logarithmic likelihood ratio test | **<0.001** | | | **<0.001** | | |

^1^Model I Adjust for None.

^2^Model II Adjust for: sex, age, family poverty income ratio, race, education, hypertension, Marital status, Alcohol, Arthritis, Congestive Heart Failure, Coronary Heart Disease, Angina, Heart Attack, Stroke, and HDL cholesterol.

^3^OR: Odds ratio.

^4^95% CI: 95% confidence interval.

Reply to Reviewers #2

Dear Reviewer,

Thank you for dedicating your time to review our manuscript and for providing positive feedback along with effective suggestions.

We are grateful for your detailed comments, which have greatly facilitated the revision of our manuscript. We earnestly hope that our responses have adequately addressed each of your concerns. In this letter, we systematically address each of your comments and provide our detailed responses.

We sincerely hope that our revised manuscript will meet with your approval.

Sincerely,

The authors

**Major comments:**

1. Page 10 – Could you please clarify the rationale behind recommending at least 12 alcoholic drinks per year, as this represents a low threshold (equivalent to approximately one drink per month) and does not align with standard alcohol consumption categories? There are international guidelines on alcohol consumption that categories alcohol intake, which could be considered to analyse further the impact on ABSI concerning various levels of alcohol intake, if data is available from the dataset.

**Author's Response:**

Thank you for your insightful comment. The threshold of more than 12 alcoholic drinks in the past year was based on the original coding scheme provided by the NHANES dataset. We acknowledge that this represents a relatively low level of alcohol consumption and may not fully align with international drinking categories. This limitation has now been clarified in the manuscript.

**Modifications:**

Line 434-442

Second, although we adjusted for a range of potential confounding factors—including age, gender, race, and other sociodemographic and clinical variables—residual confounding cannot be entirely excluded. In particular, alcohol consumption was categorized based on the NHANES coding scheme, using a threshold of more than 12 alcoholic drinks per year. We acknowledge that this definition reflects a relatively low level of intake and does not align with international guidelines, which may limit the interpretability of alcohol-related findings. Unmeasured or unaccounted-for variables may also influence the observed relationship.

2. Table 1 - The total number of various baseline characteristics, including education level, marital status, alcohol consumption, high blood pressure, arthritis, coronary heart disease, congestive heart failure, angina, heart attack, and stroke, does not equate to the cohort size (34,693 participants). Please verify the calculations for accuracy and clarify any discrepancies arising from missing data, subgroup exclusions, or reporting errors, ideally by including a footnote or explanation in the methods section.

**Author's Response:**

Thank you for your valuable comment. We have re-checked the data and confirmed its accuracy. Regarding missing values, we handled them as follows: if the proportion of missing data for a given variable exceeded 20%, that variable was excluded from the analysis. For categorical variables, missing values were imputed using the mode. For continuous variables, if the data were normally distributed, we used the mean for imputation; if not, we used the median. These imputation strategies have been described in the Methods section.

**Modifications:**

Line 53-127

Missing data were handled as follows: variables with more than 20% missing values were excluded from the analysis. For categorical variables, missing values were imputed using the mode (i.e., the most frequent category). For continuous variables, if normally distributed, the mean was used for imputation; otherwise, the median was applied.

3. In lines 166-175, please include the reference to Table 2 when the statistics for Model 1 and Model 2 are first mentioned.

**Author's Response:**

Thank you for your helpful suggestion. We have added a reference to Table 2 when Model 1 and Model 2 are first mentioned to improve clarity and guide the reader to the relevant statistical results.

**Modifications:**

Line 208-218

The results revealed a clear inverse L-shaped curve relationship between ABSI and diabetes (Fig 2). According to the threshold effect analysis, the inflection point for ABSI was determined to be 9.54. The two-part linear regression model demonstrated that when ABSI is less than or equal to 9.54, each unit increase in ABSI is associated with an 87% increased likelihood of diabetes (OR = 1.87, 95% CI: 1.72, 2.05, P < 0.001) (Table 2)

4. Lines 248-251 report a percentage increase in ABSI correlating with diabetes risk. Please specify which table or figure supports this finding to ensure clarity and traceability of the reported correlation.

**Author's Response:**

Thank you for your helpful suggestion. We have revised the manuscript to specify that the reported correlation between ABSI and diabetes risk is supported by Figure 2 and Table 2, in order to enhance clarity and traceability.

**Modifications:**

Line 283-284

In this study, we observed a significant positive correlation between ABSI and diabetes, as demonstrated in Figure 2 and Table 2.

5. Was there further analysis to explore the correlation between ABSI and lifestyle factors (e.g. sleep patterns, stress, and exercise frequency), as well as clinical characteristics (e.g. insulin resistance, beta-cell dysfunction, and other hormonal markers)? Since these factors were highly emphasized in the discussion, further investigation is necessary.

**Author's Response:**

We appreciate the reviewer’s observation. While we discussed lifestyle and clinical mechanisms in the context of existing literature, these factors (e.g., stress, beta-cell dysfunction) were not available in the dataset and could not be directly analyzed. We have revised the Discussion to clarify that these interpretations are based on prior studies.

**Modifications:**

Line 333-335

It should be noted that the proposed mechanisms involving lifestyle factors and clinical characteristics are based on findings from previous studies and were not directly assessed in our analysis.

6. The authors reference a rural Chinese study showing similar predictive effectiveness of ABSI for diabetes risk. Could the authors clarify how their findings compare, notably whether ABSI demonstrates superior predictive accuracy (e.g., higher AUC or sensitivity) over other indices like BMI in this or the Chinese cohort?

**Author's Response:**

Thank you for this valuable comment. We have revised the Discussion to clarify that the referenced rural Chinese study observed a significant association between ABSI and diabetes, but reported relatively lower predictive accuracy for ABSI compared to WHtR and BRI. In contrast, our study found a stronger association between ABSI and diabetes risk. We also clarify that our analysis did not include direct performance comparisons between ABSI and other anthropometric measures. This clarification has been added to avoid potential misinterpretation of the cited evidence.

**Modifications:**

Line 293-302

It is worth noting that a rural Chinese study also identified a significant association between ABSI and diabetes risk, although ABSI demonstrated relatively lower predictive accuracy compared to other anthropometric indices such as WHtR and BRI, with an AUC of 0.61 in both men and women. In contrast, our study found a stronger association, with individuals in the highest ABSI quartile having a 96% higher odds of diabetes compared to those in the lowest quartile. While we did not perform a direct comparative analysis between ABSI and other indices in our cohort, the findings collectively suggest that ABSI is a relevant marker for diabetes risk in diverse populations.

7. The authors (lines 367–371) underscore the significance of this study by demonstrating that ABSI is a statistically significant predictor of diabetes risk, showing positive correlations with various covariates. However, to establish ABSI as a robust predictive tool, further elaboration will be required on its application, especially for continuous variables. Specifically, the authors should clarify how ABSI’s continuous nature is modelled (e.g. through thresholds or logistic regression) to predict diabetes risk, including details on predictive performance metrics and its added value over existing measures, given the continuous covariates in the analysis.

**Author's Response:**

As this is a cross-sectional study primarily focused on examining associations rather than predictive modeling, we did not include performance metrics such as AUC or sensitivity/specificity, nor did we compare ABSI with other anthropometric indices (e.g., BMI, WC). We have added this clarification in the Discussion section to avoid overstating the predictive utility of ABSI and to align the interpretation with the study design.

**Modifications:**

Line 405-407

These findings are consistent with our results, suggesting that ABSI may be a useful indicator associated with diabetes risk across different populations.
