# Supplementary material for: Non-linear associations of a body shape index with diabetes among adults: A cross-sectional study
Source: PLoS One. 2025 Oct 23;20(10):e0333883. doi: 10.1371/journal.pone.0333883 (PMC12548853; doi:10.1371/journal.pone.0333883)
Supplement: Attachment — Submitted filename: Response to reviewers9.3.docx [file pone.0333883.s002.docx]

Re: Response for manuscript PONE-D-25-02776 “Non-linear associations of a body shape index with diabetes among adults: a cross-sectional study”

Dear Editor, Dear reviewers

We appreciate your letter dated 3 June , 2025.

We are grateful to have received the second-round peer review comments from PLOS ONE. Our heartfelt thanks go to the editors and reviewers for their valuable time, constructive feedback, and insightful suggestions, which have been instrumental in further improving the quality and clarity of our work.

In accordance with the guidelines provided, we have submitted both a clean revised version of the manuscript and a tracked-changes version. All modifications made in response to the reviewers’ comments are clearly highlighted for ease of reference.

Enclosed with this letter is our detailed, point-by-point response to the second-round comments. We greatly appreciate the continued guidance and support, and we respectfully hope that PLOS ONE will now find our revised manuscript suitable for publication.

Sincerely,

The authors

Encl. Responses to the comments from Reviewers 2.

Reply to Reviewers 2

Dear Reviewers,

We would like to express our sincere gratitude for your thoughtful and constructive comments on our manuscript during the second round of review. Your careful evaluation and insightful suggestions have been invaluable in refining and strengthening our work.

We have carefully considered each of your comments and have made the corresponding revisions to the manuscript. A detailed, point-by-point response outlining the changes is enclosed for your review. We believe that these revisions have substantially improved the clarity, accuracy, and overall quality of our study.

We are truly appreciative of the time and effort you have devoted to reviewing our work, and we thank you again for your valuable contributions.

Sincerely,

The authors

**Major comments:**
1. The number within the arthritis subgroup should be rechecked for accuracy, as prior concerns remain a mismatch between reported subgroup counts and the overall cohort size (34,693).

**Author's Response:**

We sincerely thank the reviewer for carefully checking the subgroup counts. Upon re-examination, we identified that the discrepancy was due to an error in reporting the number of participants in the Missing category for the arthritis variable. In our original submission, 100 individuals were inadvertently omitted from the Missing row. We have now corrected this error. The updated counts and percentages have been revised accordingly in the table and manuscript.

We appreciate the reviewer’s attention, which helped us ensure the accuracy and consistency of the data presentation.

**Modifications:**

**Table 1**

| **Arthritis,**  **n (%)** | **Overall** | | **Tertile 1** | **Tertile 2** | **Tertile 3** | **Tertile 4** |
| --- | --- | --- | --- | --- | --- | --- |
| No | | 25980  (74.89%) | 7079 (81.65%) | 6715 (77.43%) | 6337 (73.05%) | 5849 (67.41%) |
| Yes | | 8544 (24.63%) | 1552 (17.90%) | 1918 (22.12%) | 2294 (26.45%) | 2780 (32.04%) |
| Missing | | 169 (0.48%) | 39 (0.45%) | 39 (0.45%) | 43 (0.50%) | 48 (0.55%) |

2. It is advised to include a brief contextual statement on U.S. obesity prevalence among other population subgroups in the introduction or discussion sections. This would help establish the representativeness of the NHANES cohort for international readers and emphasise the importance of the findings related to obesity-associated diabetes risk in the American adult population.

**Author's Response:**

We appreciate the reviewer’s valuable suggestion to provide additional context on U.S. obesity prevalence. Specifically, we now highlight that more than 40% of U.S. adults are classified as obese, with the highest prevalence observed among non-Hispanic Black and Hispanic adults. This addition underscores the substantial public health burden of obesity in the United States and helps clarify the representativeness of the NHANES cohort for international readers.

**Modifications:**

Line 58-66：

In the United States, obesity remains a major public health concern, with more than 40% of adults classified as obese [11, 12]. Marked disparities exist across population subgroups, with prevalence approaching 50% among non-Hispanic Black adults and 45% among Hispanic adults, compared with lower rates in non-Hispanic White and Asian populations [11, 12]. These patterns underscore both the high burden of obesity in the U.S. and the representativeness of the NHANES cohort for evaluating obesity-related diabetes risk [11, 12]. Given the high prevalence of obesity and its strong link to diabetes, accurate assessment of body fat and its distribution is essential [13].

3. Although the manuscript clearly states that the ABSI value and K-breakpoint are derived from this study, it is recommended to briefly reiterate this in the Discussion sections to prevent readers from assuming the cut-off applies to other populations without validation.
**Author's Response:**

We thank the reviewer for this important observation. In response, we have revised the Discussion section to reiterate that the ABSI cut-off (9.54) and K-breakpoint identified in our analysis were derived specifically from the NHANES cohort. We now emphasize that their applicability to other populations should be interpreted with caution and requires further external validation. This clarification is intended to prevent readers from assuming the cut-off can be directly generalized beyond the study population.

**Modifications:**

Line 355-358：

However, this ABSI cut-off and K-breakpoint were derived specifically from the present NHANES cohort, and their applicability to other populations should be interpreted with caution and requires further external validation.
